# Totally endoscopic periareolar approach for mitral valve repair. First case reported in Peru

**DOI:** 10.47487/apcyccv.v6i1.449

**Published:** 2025-02-12

**Authors:** Josías C. Ríos-Ortega, Víctor Robles-Velarde, Zoe Díaz-Chavez

**Affiliations:** 1 Cardiovascular Surgery Department, Instituto Nacional Cardiovascular - INCOR - EsSalud, Lima, Peru. Cardiovascular Surgery Department Instituto Nacional Cardiovascular - INCOR - EsSalud Lima Peru

**Keywords:** Mitral Valve Insufficiency, Minimally Invasive Surgical Procedures, Cardiac Surgical Procedures, Insuficiencia de la Válvula Mitral, Procedimientos Quirúrgicos Mínimamente Invasivos, Procedimientos Quirúrgicos Cardíacos

## Abstract

We present the case of a 39-year-old man with a history of progressive dyspnea. Transthoracic and transesophageal echocardiography revealed severe mitral valve (MV) regurgitation due to P2 flail as well as severe tricuspid valve (TV) regurgitation. The patient underwent surgical treatment, including MV repair and TV annuloplasty, performed through a totally endoscopic periareolar approach. Postoperative evolution was satisfactory.

## Resumen

Presentamos el caso de un varón de 39 años con antecedente de disnea progresiva. La ecocardiografía transtorácica y transesofágica revelaron insuficiencia mitral grave debido a un prolapso de P2, así como insuficiencia tricuspídea severa. El paciente fue sometido a tratamiento quirúrgico, que incluyó reparación de la válvula mitral y anuloplastia tricuspídea, realizadas mediante un abordaje totalmente endoscópico periareolar. La evolución posoperatoria fue satisfactoria.

## Introduction

Conventionally, median sternotomy is the gold standard for accessing cardiac structures. However, complete division of the sternum often results in chronic pain, a prolonged postoperative recovery period, and an unsightly scar. For this reason, various minimally invasive techniques have been developed to mitigate the drawbacks of full sternotomy, including mini-thoracotomy and mini sternotomy. Among these, fully endoscopic techniques cause minimal trauma to the patient and have recently gained popularity. ^1^^,^^2^ In mini-thoracotomy, rib spreading is typically performed through the 4th or 5th intercostal space, which can lead to pain due to nerve injury. In contrast, fully endoscopic techniques minimize rib spreading, reducing postoperative discomfort.

Since 2012, our hospital has implemented a minimally invasive surgery program. Here, we present the first case in our country of totally endoscopic mitral valve (MV) repair performed through a peri-areolar approach.

## Case report

**Figure 1:**
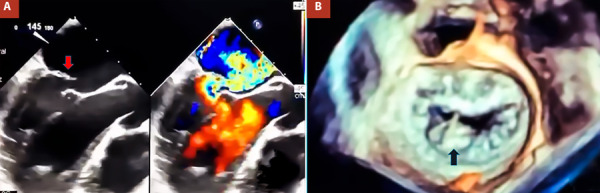
A. Pre-operative transesophageal echocardiography showing P2 flail (145o view) (red arrow). B. 3D mitral valve reconstruction showing P2 flail (black arrow).

A 39-year-old man with a history of progressive dyspnea was admitted to our institution. On admission, he reported class II dyspnea and orthopnea. On physical examination, he had rhythmic heart sounds and a grade III/VI systolic murmur at the mitral focus. Electrocardiography showed sinus rhythm. A chest X-ray revealed an increased thoracic index. Transthoracic echocardiography (TTE) identified severe MV regurgitation due to P2 flail and severe tricuspid regurgitation secondary to annular dilatation (annular diameter: 42 mm). Left ventricular ejection fraction was 54%, and right ventricular outflow tract shortening fraction was 27%. Cardiac catheterization showed no significant coronary lesions, with pulmonary artery pressure of 44/25 mmHg, wedge pressure of 24 mmHg, systemic vascular resistance of 23.9 Woods, and pulmonary vascular resistance of 2.4 Woods. Transesophageal echocardiography (TEE) confirmed these findings (Figure 1A, B). After a heart team discussion, surgical treatment was decided.

### Surgical technique

**Figure 2:**
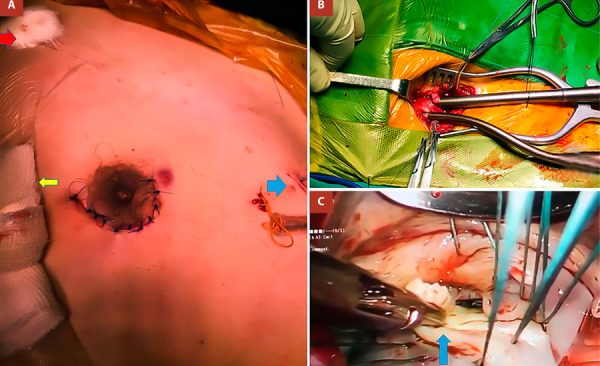
Surgical Technique. **A.** Periareolar incision, clamp port (red arrow), camera port (yellow arrow), mitral valve retractor port (blue arrow). **B.** Femoral artery and vein cannulation. **C.** Resecting P2 segment.

Endotracheal intubation was performed using a double-lumen tube. A 4 cm incision was made along the lower edge of the right areola (Figure 2A). After carefully separating the fibers of the pectoral muscle, the thorax was accessed with minimal rib separation in the 4th intercostal space using a wound retractor (Alexis®). 

For cardiopulmonary bypass (CPB), cannulation was performed via the right femoral artery and vein, along with percutaneous jugular cannulation. Vacuum-assisted drainage was used in the extracorporeal circuit (-20 to -30 cm H2O), and the caval veins were not snared (Figure 2B). A 5 mm trocar was introduced into the third intercostal space at the right mid-axillary line for the 30-degree videoscope. A transcutaneous Chitwood clamp was placed in the second intercostal space at the anterior axillary line for aortic cross-clamping (Figure 2A). 

**Figure 3:**
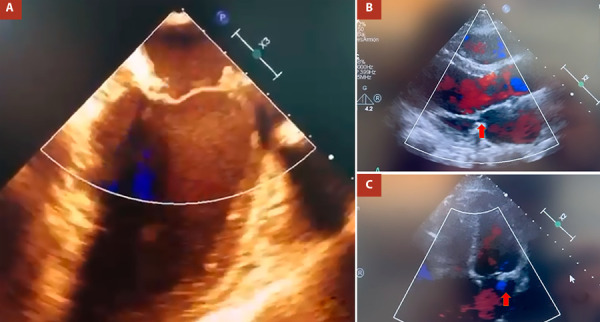
A. Postoperative transesophageal echocardiography showing no mitral valve regurgitation (180o view). B, C. Postoperative transthoracic echocardiography showing minimal mitral valve regurgitation in parasternal and 4 camera views, respectively (red arrow).

Cardioplegia was administered as a single dose of HTK Custodiol® (25 mL/kg) via the ascending aorta. A conventional left atriotomy was performed, revealing ruptured chordae and prolapse (flail) of the middle portion of the posterior leaflet (P2) with annular dilation. A triangular resection was performed, and the leaflet was sutured using 5/0 polypropylene. A 32 mm incomplete semi-rigid ring was subsequently implanted. The left atrium was closed with 4/0 polypropylene. Next, the right atrium was opened, and a 30 mm incomplete semi-rigid ring was implanted for tricuspid valve repair. An epicardial pacemaker electrode was placed before aortic unclamping. CPB and cross-clamp times were 120 and 105 minutes, respectively. Intraoperative TEE showed mild mitral and tricuspid regurgitations (Figure 3A).

The patient was successfully weaned from mechanical ventilation in the operating room, with a total blood loss of 350 mL in the first 24 hours. Postoperative evolution was satisfactory; the hospital stay was 5 days. A TTE performed before discharge showed a mitral valve with adequate gradients (mean: 2 mmHg) and mild regurgitation. After four years of follow-up, the patient remains in functional class I, with mild mitral and tricuspid regurgitation (Figure 3B, C).

## Discussion

Peru has a fragmented health system, and health inequalities are a serious problem. In this context, the social security system (EsSalud) provides coverage for approximately 30% of the population. ^3^ Although minimally invasive techniques in cardiac surgery were introduced in the 1990s, their implementation at our center-the leading hospital for cardiovascular pathology in Peru-began in the 2010s. Given the structural and resource limitations of our health system, this represented a considerable challenge.

MV repair via minithoracotomy has not been shown to reduce operative mortality compared to conventional access. However, it significantly decreases intra- and postoperative bleeding rates, as well as hospital length of stay. Additionally, the aesthetic benefits are noteworthy. ^(^^2^^,^^4^

The inclusion of videoscopy in minimally invasive approaches enables even smaller incisions with minimal rib separation, leading to reduced pain and greater aesthetic acceptance. ^(^^5^ The periareolar approach involves a small convex incision along the right areolar border, allowing the surgeon to access the heart through the 3rd, 4th, or 5th intercostal space without traumatic rib spreading. ^(^^6^

In Brazil, Oliveira *et al*. compared 21 patients who underwent right minithoracotomy with 16 patients operated on via periareolar access. No significant differences were found in incision length, cardiopulmonary bypass time, aortic cross-clamp time, hematocrit levels, chest tube drainage volume, or length of stay in the intensive care unit and hospital. However, time to extubation showed a significant difference between the right minithoracotomy and periareolar access groups (4.85 hours vs. 5.62 hours, respectively; p = 0.04). ^(^^7^

Although the periareolar approach was initially developed for male patients, it has also been successfully performed in women in several centers. In Italy, Brega *et al*. reported a series of 57 female patients who underwent a periareolar incision as a minithoracotomy approach. Among them, 87.7% (50 patients) underwent mitral valve (MV) repair, including six with associated procedures; 8.8% (five patients) underwent MV replacement, two of whom also underwent tricuspid annuloplasty; and 3.5% (two patients) underwent isolated tricuspid surgery. The cardiopulmonary bypass and aortic cross-clamp times were 123.2 ± 30.2 minutes and 101.3 ± minutes, respectively. No conversions to full sternotomy or a larger thoracotomy approach were required, and there were no in-hospital or follow-up deaths.^8^ Similar results were reported by Poffo *et al*. in a study of 214 patients (including women) in Brazil. While periareolar access has been successfully performed in female patients, it has not yet been reported in our country. ^9^
